# Syndrome coronarien aigu chez une jeune patiente: au-delà des lésions coronaires

**DOI:** 10.11604/pamj.2018.29.134.12062

**Published:** 2018-02-27

**Authors:** Aridane Cárdenes León, Lucas Robador, Antonio García Quintana, Miguel Ángel Cárdenes Santana, Pablo Felipe Bujanda Morún, Noel Lorenzo Villalba

**Affiliations:** 1 Service de Cardiologie, Hôpital Universitaire de Gran Canaria Dr Negrín, Las Palmas de Gran Canaria, Espagne; 2 Service de Radiologie, Hôpital Universitaire de Gran Canaria Dr Negrín, Las Palmas de Gran Canaria, Espagne; 3 Service de Médecine Interne, Hôpital Universitaire de Gran Canaria Dr Negrín, Las Palmas de Gran Canaria, Espagne; 4 Service de Médecine Interne et Cancérologie, Centre Hospitalier Saint Cyr, France

**Keywords:** Syndrome coronarien aigu, prothrombine G201210A, facteur anticoagulant lupique, Acute coronary syndrome, G201210A Prothrombin Gene, lupus anticoagulant factor

## Abstract

Nous exposons le cas d’une patiente de 47 ans qui présente un syndrome coronarien aigu et une probable embolie artérielle du membre inférieur droit dans l’étude duquel nous détectons la présence de la mutation du gène de la prothrombine G201210A associée à la présence de facteur anticoagulant lupique. La patiente a bénéficié d’une transplantation cardiaque avec bonne évolution clinique.

## Abstract (en)

We here report the case of a 47-year old female patient with acute coronary syndrome associated with possible arterial embolism of the right lower limb. During examination we detected G201210A Mutation of the Prothrombin Gene associated with lupus anticoagulant factor.

## Introduction

La mutation du gène de la prothrombine G20210A représente un risque de thrombophilie accentué. Traditionnellement, on a décrit une augmentation importante du risque de thrombose veineuse chez ces patients; cependant, lors des dernières années, de nombreuses études qui démontrent une augmentation d’incidence de thrombose artérielle et de cardiopathie ischémique chez les porteurs de cette variante génétique ont été publiées.

## Patient et observation

Femme de 47 ans, fumeuse, sans autres antécédents personnels ni familiaux importants, sous traitement avec des contraceptifs oraux. La patiente va à l’hôpital après présentation d’un cadre de douleur centro-thoracique oppressive associé à des symptômes végétatifs au repos. À son arrivée, cliniquement symptomatique avec un examen physique anodin. L’électrocardiogramme présentait un rythme sinusal avec une supra-dénivellation du ST, sur II, III, aVF, V5-V6 et sur des dérivations postérieures et une chute sur aVL, V1-V2. Analytiquement, il fallait souligner l’augmentation enzymatique importante, avec une créatine-kinase (CK) maximale de 1850U/L et une troponine T (TnThs) maximale de 1079ng/ml. Dans l’échocardiogramme transthoracique on objective la présence d’un léger dysfonctionnement ventriculaire aux dépens d’affectation des différents territoires coronariens, principalement dans la région apicale et dans le cathétérisme cardiaque on constate la présence de micro embolies coronariennes au niveau de l’arbre coronarien gauche: sur le tiers distal de l’artère descendante antérieure, artère diagonale et artère obtuse marginale (branche d’artère circonflexe). Dans la ventriculographie on confirme l’akinésie antéro-apicale.

**Figure 1:**
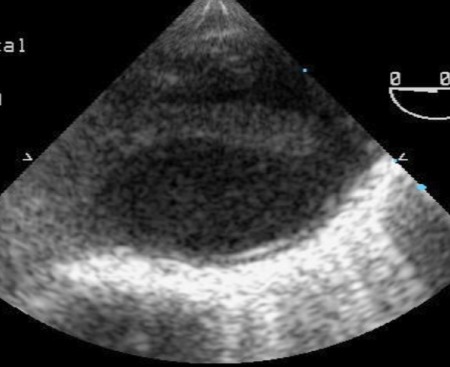
Image de thrombus mural intraaortique digitiforme objectivé dans ETE

**Figure 2:**
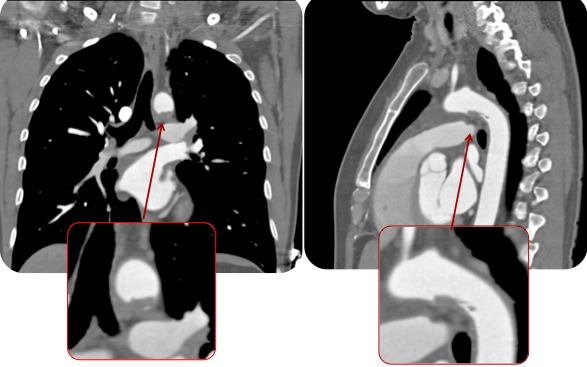
TAC cardiaque; vision du thrombus aortique mural en 7a: coupe axiale, 7b: coupe coronale et sagittale

**Figure 3:**
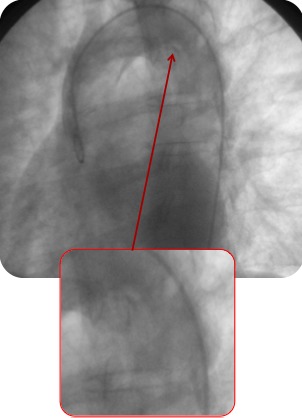
Aortographie: défaut de réplétion sur l’emplacement du thrombus aortique

Évolution clinique favorable sous traitement médical en restant asymptomatique. Compte tenu du contexte clinique et des découvertes dans les examens complémentaires, on réalise une étude d’extension pour exclure un éventuel foyer emboligène. Dans le holter on ne documente pas d’évènements arythmiques ni d’autres découvertes pathologiques. Cependant dans l’échocardiogramme trans-oesophagien au niveau de la crosse aortique distale, on objective une image suggestive de thrombus mobile digitiforme adhéré à la paroi, de quelques 14 mm de long (Figure 1). Le reste de l’aorte semble être assez saine, sans athéromatose ni image de dissection sur tout le trajet visualisé. La présence du thrombus mural intra-aortique mentionné est confirmée par le scanner cardiaque (Figure 2) et dans l’aortographie (Figure 3).

On réalise une étude de thrombophilie après plusieurs semaines (l’étude mentionnée peut être faussée pendant la phase aigue de l’évènement ischémique, c’est pourquoi il est recommandé d’attendre au mois 2-3 mois), en objectivant que la patiente est porteuse de la mutation du gène de la prothrombine G20210A, ainsi que la présence du facteur anticoagulant lupique, ce dernier lors de deux déterminations séparées par un intervalle de 6 semaines. L’étude familiale de thrombophilie détecte trois cousins présentant la mutation du facteur V de Leyden (2 d’entre eux sous traitement préalable avec sintrom après avoir présenté des thromboembolismes veineux). La patiente abandonna l’hôpital avec un traitement antiagrégant (adiro 100mg), anticoagulant oral (sintrom pendant six mois au moins), bétabloquant, faibles doses d’un IECA et statine. On recommanda l’arrêt de la prise de contraceptifs oraux. Pendant les mois postérieurs, la patiente présente un cadre de claudication intermittente du membre inférieur droit. On réalise une angio-TAC, qui met en évidence l’occlusion de l’artère fémorale droite, avec une circulation co-latérale abondante, cette découverte est interprétée comme en rapport avec un éventuel évènement thromboembolique artériel.

La résonance magnétique cardiaque réalisée six mois après l’évènement ischémique décrit la présence d’un thrombus de quelques 10mm de long, avec une certaine réduction de taille par rapport aux études préalables. Après l’introduction d’un contraste, on n’observe pas de captation de la masse, ce qui renforce, plus encore, l’hypothèse de thrombus mural intra-aortique et qui exclut totalement le soupçon de néoplasie endothéliale. Un an après l’évènement coronarien, l’échocardiogramme transthoracique décrit un rétablissement complet de la fonction ventriculaire, la patiente étant totalement asymptomatique du point de vue cardiologique. Compte tenu de la persistance du thrombus mural intra-aortique dans la résonance magnétique, la mutation du gène de la prothrombine G20210A et les antécédents emboliques (embolismes coronariens et embolie artérielle probable du membre inférieur droit), on décide un suivi clinique strict, traitement anticoagulant (sintrom) indéfiniment et on arrête l’adiro un an s’étant écoulé depuis l’évènement coronarien aigu.

## Discussion

La mutation du gène de la prothrombine G20210A est associée à des niveaux plus élevés de prothrombine et complexes thrombine-antithrombine III, ce qui confère un état clinique d’hypercoagulabilité. Il existe une grande évidence en rapport avec l’augmentation de l’incidence de thrombose veineuse chez les porteurs de cette variante génétique. Cependant, malgré des controverses dans certaines recherches, on a observé en outre une association importante entre l’allèle 20210A et l’infarctus du myocarde, spécialement chez des femmes jeunes (âgées de moins de 55 ans) [1]. De nombreuses études décrivent une association importante entre cette mutation et l’AVC chez les jeunes patients, la thrombose artérielle coronarienne, cérébrale ou périphérique chez des patients sans facteurs de risque classiques. L’administration concomitante de contraceptifs oraux chez des patientes ayant ces mutations prothrombotiques semble augmenter le risque d’évènements thromboemboliques [2].

Les découvertes décrites dans diverses recherches suggèrent que l’allèle prothrombine 20210A représente un facteur de risque héréditaire pour le syndrome coronarien aigu chez les patients ayant des antécédents d’infarctus du myocarde qui ont une étendue limitée de la maladie coronarienne dans l’angiographie (maladie coronarienne mono-vasculaire ou des lésions non importantes) et qui sont dépourvues d’altérations métaboliques ou de facteurs de risque acquis (cependant, plusieurs études décrivent un risque significativement majeur d’infarctus du myocarde quand la variante prothrombine G20210 coexiste avec d’autres facteurs de risque cardiovasculaire, comme le tabagisme et l’hypercholestérolémie) [3,4]. Selon ce qui est décrit, si la mutation mentionnée est importante pour la cardiopathie ischémique, il est probable qu’elle le soit dans des conditions cliniques dans lesquelles la thrombose est le fait fondamental (angine instable, infarctus aigu du myocarde), mais pas dans les angines stables où l’artériosclérose est le facteur pathogénique prédominant. La recherche de marqueurs phénotypiques, comme l’âge et le degré de maladie de l’artère coronarienne pourrait représenter une exigence préalable générale importante pour de futures études génétiques afin d’obtenir des informations d’utilité clinique dans cet aspect. On doit donc soupçonner ce type de mutations prothrombotiques chez des jeunes patients de moins de 55 ans (spécialement les femmes), avec des antécédents familiaux de cardiopathie ischémique, sans facteurs de risque cardiovasculaire, qui ont présenté un syndrome coronarien aigu, surtout chez ceux ayant une maladie coronarienne limitée dans l’angiographie.

En ce qui concerne les thrombus muraux intra-aortiques, ceux-ci sont fréquemment décrits dans la littérature. Dans la plupart des cas, ils sont associés à l’artériosclérose généralisée, mais il existe de nombreuses occasions dans lesquelles nous trouvons ces thrombus en association avec des aortes totalement saines, dans ces cas nous devons étudier d’autres causes : anévrismes, dissection, état d’hypercoagulabilité, etc. La zone autour de l’artère subclavière gauche semble être un des emplacements prédisposés pour la formation desdits thrombus aortiques. Étant donné l’emplacement de ce thrombus, notre soupçon principal est la présence d’un thrombus ou caillot dans les cavités gauches (non objectivé dans les examens d’image) qui se serait détaché vers le sinus coronarien gauche et postérieurement dans tout l’arbre coronarien gauche, en provoquant ainsi des thromboembolismes à différents niveaux. Cependant on ne peut pas écarter absolument l’embolisation du thrombus situé juste distal à la sortie de la subclavière gauche, bien que nous le considérons peu probable par son emplacement chez une patiente qui ne présente pas d’insuffisance aortique importante ni un autre type de pathologies concomitantes qui produiraient un flux rétrograde diastolique intense à ce niveau.

La manipulation optimale des thrombus muraux n’est pas encore totalement définie. L’anticoagulation serait indiquée après avoir présenté des évènements emboliques, spécialement chez des patients souffrant de variations génétiques prothrombotiques (chez ces derniers de manière indéfinie). Certains auteurs recommandent la thrombectomie chirurgicale au lieu du régime anticoagulant chez des jeunes patients, en présence d’un grand thrombus hypermobile et des patients ayant des évènements emboliques récurrents si les risques chirurgicaux sont acceptables [4]. Le traitement à l’aide de stent endovasculaire fournit une nouvelle option thérapeutique moins invasive dans le traitement de la thrombose aortique thoracique mobile symptomatique; cependant, son rôle dans la fixation de la thrombose aortique quant à l’évolution à long terme n’a pas encore été établi [4-6]. Dans notre cas l’anticoagulation de manière indéfinie est indiquée chez une patiente porteuse de cette mutation qui de plus a présenté deux évènements thromboemboliques: thromboembolisme coronarien qui provoqua un infarctus du myocarde et un thromboembolisme artériel périphérique probable dans le membre inférieur droit. Si la patiente présentait des épisodes emboliques récurrents et la persistance à long terme de ce thrombus, on devrait évaluer la possibilité de réaliser une thrombectomie chirurgicale ou un traitement avec un stent endovasculaire. Dans la littérature, la plupart de cas décrits présentent une association de plusieurs troubles de la coagulation : carence en protéine C ou S, facteur V et présence de la mutation du gène de la prothrombine G201210A avec une présentation clinique aussi variée. Les résultats décrits lors de l’angiographie sont aussi variés ayant de la présence d’un thrombus à la présence de lésions non importantes [7-10].

## Conclusion

Une étude de thrombophilie sera réalisée chez des jeunes patients, sans facteurs de risque, qui auraient présenté un syndrome coronarien aigu, spécialement chez ceux ayant une maladie coronarienne limitée dans l’angiographie.
